# The American Institute

**Published:** 1883-11

**Authors:** 


### THE AMERICAN INSTITUTE.

The *United States Medical Investigator* has found out at last that the Institute is an arrant humbug. This must be very patent when the *Investigator* feels compelled to write of it. The *Investigator* is not a *severe* critic. It says: "Taken altogether, the question will arise, would not a little more medicine in the Institute and less politics, potencies, and polemics [and whisky], call out more interest?"

The Institute talks a great deal about medical subjects, but the talk is generally poor and the "medicine" worse. A few hours' study of the *Organon* would be serviceable.

